# Volatilome Characterization of the Flat White Onion of Fara Filiorum Petri (*Allium cepa* L.) and Its Differentiation from Other Italian White Onions by HS-SPME-GC-MS

**DOI:** 10.3390/molecules31193393

**Published:** 2026-09-24

**Authors:** Samantha Reale, Lorenzo Marsili, Marco Di Santo, Luciano Di Martino, Alessandra Biancolillo, Angelo Antonio D’Archivio

**Affiliations:** 1 Department of Physical and Chemical Sciences, University of L’Aquila, Via Vetoio, Coppito, 67100 L’Aquila, Italy; 2 Majella Seed Bank-Parco Nazionale della Majella, Via Badia 28, 67039 Sulmona, Italy

**Keywords:** Flat White Onion of Fara Filiorum Petri, *Allium cepa* L., volatile organic compounds (VOCs), HS-SPME-GC-MS, volatilome, principal component analysis, food authentication, agrobiodiversity

## Abstract

The Flat White Onion of Fara Filiorum Petri is a traditional Italian landrace that represents the agrobiodiversity and gastronomic heritage of the Abruzzo region. However, its volatile composition has not yet been characterized. In this study, the volatile profile of 31 samples of Flat White Onion of Fara Filiorum Petri and other (53) Italian white onions was investigated by headspace solid-phase microextraction coupled with gas chromatography–mass spectrometry (HS-SPME-GC-MS). A total of 51 volatile organic compounds (VOCs) were tentatively identified as characteristic of onion aroma. Principal component analysis (PCA) revealed a characteristic volatilomic fingerprint for the Flat White Onion of Fara Filiorum Petri and highlighted a high degree of consistency between the two harvest years, indicating that seasonal variability mainly affected the relative abundance of selected VOCs while preserving the overall volatile profile. Authentication was subsequently investigated using Soft Independent Modeling of Class Analogy (SIMCA). The optimal model correctly recognized 88.9% of Fara samples in the external test set while rejecting all non-Fara onions, achieving 100.0% specificity. These findings demonstrate that HS-SPME-GC-MS combined with chemometric analysis provides an effective approach for the characterization and authentication of the FlatWhite Onion of Fara Filiorum Petri, supporting the conservation of this Italian landrace.

## 1. Introduction

Onion (*Allium cepa* L.) is one of the oldest and most widely cultivated vegetable crops worldwide and represents an essential ingredient in many traditional diets due to its sensory, nutritional, and health-promoting properties [1,2]. Besides being an important source of carbohydrates, vitamins, minerals, and phenolic compounds, onions are particularly appreciated for their characteristic aroma, which strongly influences consumer preference and culinary applications. The distinctive flavour of onion is mainly associated with a complex mixture of volatile organic compounds (VOCs), among which sulfur-containing compounds play a predominant role [3]. These molecules are responsible for the typical pungent notes of fresh onion and are also involved in many of its biological activities, including antioxidant, antimicrobial, and anti-inflammatory effects [1].

Commercial onions are commonly classified according to bulb colour into yellow, red, and white varieties, each characterized by distinct sensory and phytochemical traits. White onions generally exhibit lower concentrations of anthocyanins and a milder, sweeter taste than red and yellow cultivars, making them particularly appreciated for fresh consumption and for culinary preparations in which a delicate flavour profile is desired [4,5]. Despite their economic and gastronomic importance, white onion landraces have received considerably less scientific attention than coloured cultivars, especially regarding their volatile composition and aroma determinants.

The characteristic aroma of onion originates from a specialized sulfur metabolism. In intact tissues, flavour precursors are present mainly as S-alk(en)yl-L-cysteine sulfoxides (ACSOs), which are compartmentalized separately from the enzyme alliinase. When tissue is disrupted by cutting, crushing, or processing, alliinase hydrolyses ACSOs to produce unstable sulfenic acids, which are rapidly converted into reactive intermediates including thiosulfinates and (via lachrymatory factor synthase acting on 1-propenyl sulfenic acid) the lachrymatory factor propanethial S-oxide responsible for tearing [6].

These unstable compounds rapidly decompose or rearrange to form a wide variety of secondary sulfur volatiles—for example sulfides, disulfides, trisulfides, thiosulfonates, and thiophenes—that largely determine the final aroma profile of onion products [7,8].

Although sulfur compounds dominate the aroma profile, onion volatilomes also contain numerous non-sulfur constituents, including aldehydes, alcohols, ketones, esters, furans, and organic acids. Their relative abundance depends on genotype, agronomic practices, maturity stage, environmental conditions, post-harvest handling, and processing treatments [9,10]. Recent investigations have shown that cooking, storage conditions, drying processes, irradiation, and fresh-cut operations substantially modify the volatile composition of onions, generally decreasing the abundance of reactive sulfur compounds while promoting the formation of thermally generated volatiles and lipid-derived compounds [5,11].

Because of the complexity and high reactivity of onion volatiles, their characterization remains analytically challenging. Over the years, a variety of extraction and separation approaches have been employed, including steam distillation, solvent extraction, supercritical fluid extraction, direct headspace analysis, and multidimensional gas chromatography [12,13,14]. Among these methodologies, headspace solid-phase microextraction coupled with gas chromatography–mass spectrometry (HS-SPME-GC-MS) has become one of the most widely adopted techniques because it provides high sensitivity, minimal sample preparation, and reduced artefact formation, making it particularly suitable for the analysis of highly volatile sulfur compounds [15,16]. More recently, the application of advanced metabolomic approaches, comprehensive two-dimensional gas chromatography, ion mobility spectrometry, and machine learning techniques has considerably expanded the knowledge of onion volatilomes and improved the discrimination of varieties and processing conditions [4,5,17].

Despite the considerable body of literature devoted to the characterization of onion volatile compounds, most studies have focused on commercial cultivars or pigmented onion varieties [9], while comparatively few investigations have addressed the volatile profiles of local onion landraces [18]. Traditional genotypes may possess unique metabolic traits arising from long-term adaptation to specific pedoclimatic conditions and cultivation practices, potentially leading to distinctive aromatic fingerprints. The characterization of the volatile composition of local onion ecotypes is therefore important not only for understanding their sensory attributes but also for supporting their valorisation, conservation, and potential geographical differentiation.

The Flat White Onion of Fara Filiorum Petri (Figure 1) is an ancient Italian landrace cultivated exclusively in the municipality of Fara Filiorum Petri, in the Abruzzo region of central Italy. Locally known as “piattona” because of its characteristic flattened bulb shape, this onion is distinguished by its white tunic, remarkable sweetness and delicate flavour. Historical records suggest that its cultivation dates back to at least the fourteenth century and that the variety has been maintained over the centuries owing to its adaptation to the specific pedoclimatic conditions of the Foro Valley. Today, the Flat White Onion of Fara Filiorum Petri is recognized as a Traditional Agri-food Product and a Slow Food Presidium, representing an important component of the regional agricultural biodiversity and gastronomic heritage [19].

However, despite its historical, cultural, and gastronomic importance, no scientific information is currently available regarding the volatile composition of the Flat White Onion of Fara Filiorum Petri. Therefore, the aim of the present study was to comprehensively characterize its volatile profile by means of HS-SPME-GC-MS and exploratory data analysis, providing new insights into the aromatic composition of this traditional Italian onion landrace and contributing to its scientific characterization and valorisation.

## 2. Results and Discussion

### 2.1. HS-SPME-GC-MS

The volatile composition of the investigated white onion samples was characterized by HS-SPME-GC-MS, leading to the tentative identification of 51 volatile organic compounds (VOCs) (Table 1). Compound tentative identification was achieved by combining mass spectral matching with retention index comparison using a C7–C40 n-alkane series. The tentatively identified VOCs mainly belonged to the chemical classes typically associated with onion aroma, including sulfur-containing compounds, aldehydes, terpenes, esters and furan derivatives [3].

Since relative abundances were calculated from peak areas normalized to the total ion chromatogram (TIC), the reported values do not represent absolute concentrations but rather the relative contribution of each VOC to the overall volatile profile. Therefore, the following discussion focuses on differences in the relative distribution of VOCs among onion samples. An example of a typical HS-SPME-GC-MS chromatogram is shown in Appendix A.

As expected for *Allium cepa* L., sulfur-containing compounds represented the predominant fraction of the volatilome in all samples. Among them, propyl disulfide (**23**) showed the largest mean relative contribution within almost all onion types, accounting for approximately 43% and 36% of the total volatile fraction in the Flat White Onion of Fara Filiorum Petri (FFP) harvested in 2024 and 2025, respectively, 42% in white onion from Calabria region (CAL_2025), 35% in white onion from Giarratana (GIARR_2025), and about 56% in both white onion from Margherita di Savoia (MDS_2025) and the white onion from Margherita di Savoia Protected Geographical Indication (MDS_PGI_2025).

Interestingly, the samples harvested in 2026 showed a different descriptive sulfur-containing volatile pattern. In MDS_PGI_2026 and the onion labelled with generic Italian origin (ITA_2026), the mean relative abundance of propyl disulfide was ca. 15% and 18%, respectively, whereas mean values in the remaining onion groups ranged from approximately 35% to 56%. The overall sulfur contribution was ca. 78% and 75% of the tentatively identified volatile fraction, respectively. For comparison, sulfur compounds accounted for more than 90% of the volatile fraction in several of the other commercial onion groups, while mean contributions of 84% and 80% were observed in FFP_2024 and FFP_2025, respectively. These descriptive differences indicate substantial variation in the sulfur-containing fraction among the investigated onion groups and harvest years. Within the 2024–2025 samples, the mean sulfur contribution observed for FFP was 84% in FFP_2024 and 80% in FFP_2025. These values are reported as descriptive differences and are not intended to imply statistical significance or a direct relationship with sensory perception.

For *syn*-propanethial-*S*-oxide (peak **2**), the well-known onion lachrymatory factor, a mean relative abundance of ca. 1% was observed in FFP_2024 and ca. 2% in MDS_2025. The corresponding mean values were approximately 4% in FFP_2025 and CAL_2025, 3% in MDS_PGI_2025 and GIARR_2025, 15% in MDS_PGI_2026, and 14% in ITA_2026. Thus, the investigated groups showed considerable descriptive variation in the relative contribution of this compound. Its lower relative abundance in FFP2024 may be consistent with the traditionally reported mild sensory profile of this landrace, although no direct correlation between VOC relative abundance and sensory perception can be established from the present data.

The difference observed between FFP_2024 and FFP_2025 further indicates that the relative contribution of this single compound varied between the two investigated harvest years and should therefore not be considered, on its own, a defining feature of FFP.

Terpenes showed marked descriptive differences among the investigated onion groups. In FFP, the mean cumulative relative areas of terpenes were approximately 5% and 3% in the 2024 and 2025 harvests, respectively. In the other onion groups, the corresponding mean values were below 0.5%, and no terpenes were detected at all in GIARR_2025. The terpene fraction of FFP comprised β-pinene (**11**), β-myrcene (**12**), 3-carene (**15**), *o*-cymene (**16**), limonene (**17**), *cis*-β-ocimene (**18**), γ-terpinene (**19**), and α-terpinolene (**20**), providing a broader terpene profile in the FFP samples under the analytical conditions adopted. Since monoterpenes are commonly associated with fresh, citrus-like, floral, herbal, and resinous aroma notes, this descriptive compositional pattern may be relevant to the sensory characteristics traditionally attributed to FFP [20,21]; however, no direct sensory inference can be drawn from the present data.

Aliphatic aldehydes represented another important group of VOCs. Hexanal (**3**), octanal (**14**), nonanal (**22**) and decanal (**33**) were detected in all investigated white onions, with nonanal and decanal being the most abundant representatives of this class. Aldehydes are commonly generated through lipid oxidation pathways and are generally associated with fresh, green and fatty sensory notes [21,22]. Their mean relative contribution varied considerably among onion groups. Relevant cumulative aldehyde contents were observed in the 2026 samples, ITA_2026 (24%) and MDS_PGI_2026 (22%), in opposition to FFP_2025 (14%), CAL_2025 (2%), GIARR_2025 and MDS_PGI_2025 (4%), and MDS_2025 and FFP_2024 (7%). Nonanal was the predominant aldehyde in most samples, reaching approximately 11% in both MDS_PGI_2026 and ITA_2026 and 9% in FFP_2025. These values are presented as descriptive differences among the investigated groups.

Among the tentatively identified non-sulfur compounds, 2-hexyl-5-methyl-3(2*H*)-furanone (**44**) and 2-octyl-5-methyl-3(2*H*)-furanone (**51**) were consistently detected in FFP samples, reaching mean relative abundances of 2.7% and 1.4%, respectively, in the 2024 harvest and 1.8% and 1.4% in the 2025 harvest. In contrast, these compounds were absent or detected only at trace levels in the other onion varieties investigated. Notably, 2-hexyl-5-methyl-3(2*H*)-furanone has been referred to in the literature as the “onion furanone” [23]. Both compounds belong to the class of alkyl-substituted furanones whose biological significance in onions remains poorly understood. The recurrent detection of these compounds in FFP samples from both investigated harvest years, together with their low or non-detectable mean relative contributions in most of the other onion groups, indicates that they may represent potentially distinctive features of the volatile profile of the Flat White Onion of Fara Filiorum Petri. Together with the terpene fraction, their occurrence in both FFP harvest years identifies these compounds as candidates of interest for further investigation of the characteristic volatile profile of this landrace. However, because only two harvest years were investigated and no formal univariate statistical comparison was performed, these observations should be regarded as descriptive and do not establish that the compounds are intrinsic cultivar markers or independent of environmental and post-harvest effects.

Taken together, these results highlight a distinctive volatile profile for the Flat White Onion of Fara Filiorum Petri. Although sulfur-containing compounds remained the predominant constituents the mean volatile profile of FFP was characterized by sulfur and terpene contributions, respectively. The simultaneous occurrence of sulfur compounds, terpenes, and alkyl-substituted furanones contributed to a compositionally diverse volatile profile. In particular, the observed relative contributions of these chemical classes and compounds provide a descriptive chemical characterization of FFP and identify features that may warrant further investigation in relation to the sensory properties traditionally attributed to this landrace. However, these observations are based on relative VOC abundances and should not be interpreted as direct evidence of aroma intensity.

Nevertheless, based on previously reported associations between these tentatively identified compounds and their sensory properties, the observed volatile composition appears compatible with the traditionally described mild and aromatic character of FFP. As no sensory evaluation was performed in the present study, this interpretation should be regarded as a hypothesis, and dedicated sensory analyses and odor-activity measurements would be required to establish a direct relationship between the volatile profile and the sensory characteristics of FFP.

**Table 1:** Volatile composition of the investigated white onion samples determined by HS-SPME-GC-MS. For each compound, the peak number (#), retention time (RT, min), tentative identification, experimental (Exp) and literature (Lit) retention indices, and mean relative peak area (%) ± standard error (SE) are reported (calculated from the number of GC-MS chromatograms indicated in parentheses (*n*) for each sample). Relative peak areas were calculated as the percentage contribution of each compound to the total area of the 51 tentatively identified volatile compounds. Sample codes: FFP, Flat White Onion of Fara Filiorum Petri harvested in 2024 and 2025; MDS_PGI, White Onion from Margherita di Savoia PGI harvested in 2025 and 2026; MDS, White Onion from Margherita di Savoia harvested in 2025; CAL, White Onion from Calabria harvested in 2025; GIARR, White Onion from Giarratana harvested in 2025; ITA, White Onion from Italy harvested in 2026.

			Retention Index		FFP 2024 (*n* = 19)	FFP 2025 (*n* = 12)	MDS_PGI 2025 (*n* = 12)	MDS_PGI 2026 (*n* = 9)	MDS 2025 (*n* = 11)	CAL 2025 (*n* = 5)	GIARR 2025 (*n* = 11)	ITA 2026 (*n* = 5)
#	RT (min)	Compound	Exp ^a^	Lit ^b^	Mean A% ±SE	Mean A% ±SE	Mean A% ±SE	Mean A% ±SE	Mean A% ±SE	Mean A% ±SE	Mean A% ±SE	Mean A% ±SE
**1**	3.73	Disulfide, dimethyl	-	746 ± 6	0.71 ± 0.11	0.85 ± 0.37	0.21 ± 0.04	0.06 ± 0.06	0.15 ± 0.04	0.13 ± 0.05	0.19 ± 0.03	n.d.
**2**	4.3	*syn*-Propanethial-*S*-oxide	-	821 ± n/a	1.09 ± 0.14	4.35 ± 0.82	3.04 ± 0.47	14.93 ± 1.94	2.09 ± 0.70	3.78 ± 0.63	3.08 ± 0.69	13.50 ± 2.41
**3**	5.04	Hexanal	800	800 ± 2	1.44 ± 0.18	1.86 ± 0.49	0.33 ± 0.08	2.11 ± 0.41	0.21 ± 0.03	0.77 ± 0.18	0.29 ± 0.04	2.45 ± 0.38
**4**	5.81	2-Pentenal, 2-methyl-	828	837 ± 9	0.83 ± 0.09	0.85 ± 0.20	1.51 ± 0.22	3.75 ± 0.50	0.93 ± 0.37	2.57 ± 0.63	0.67 ± 0.24	4.44 ± 0.95
**5**	7.12	Thiophene, 2,5-dimethyl-	875	878 ± 11	0.10 ± 0.03	0.27 ± 0.11	0.20 ± 0.02	2.17 ± 0.61	0.22 ± 0.03	0.20 ± 0.03	0.17 ± 0.02	2.78 ± 1.15
**6**	7.81	Thiophene, 2,3-dimethyl-	899	896 ± 3	0.39 ± 0.03	0.93 ± 0.19	0.46 ± 0.05	1.60 ± 0.42	0.22 ± 0.07	0.37 ± 0.07	0.23 ± 0.03	1.58 ± 0.44
**7**	8.56	(*Z*)-1-Methyl-2-(prop-1-en-1-yl) disulfane	926	932 ± n/a	0.29 ± 0.03	0.54 ± 0.13	0.28 ± 0.05	0.24 ± 0.08	0.10 ± 0.01	0.09 ± 0.02	0.11 ± 0.01	0.29 ± 0.18
**8**	8.62	Disulfide, methyl propyl	928	932 ± 9	2.10 ± 0.22	1.58 ± 0.22	1.75 ± 0.42	2.37 ± 0.67	1.87 ± 0.24	1.55 ± 0.21	0.95 ± 0.13	1.55 ± 0.43
**9**	8.82	(*E*)-1-Methyl-2-(prop-1-en-1-yl) disulfane	935	940 ± n/a	0.89 ± 0.11	0.94 ± 0.18	1.08 ± 0.18	1.27 ± 0.27	0.16 ± 0.02	0.29 ± 0.04	0.52 ± 0.05	1.13 ± 0.14
**10**	9.67	Trisulfide, dimethyl	966	970 ± 7	0.33 ± 0.03	0.63 ± 0.15	0.30 ± 0.05	n.d	0.20 ± 0.03	0.09 ± 0.04	0.18 ± 0.03	n.d.
**11**	9.93	β-Pinene	975	979 ± 2	0.21 ± 0.05	0.006 ± 0.010	n.d	n.d	n.d.	0.06 ± 0.06	n.d.	n.d.
**12**	10.29	β-Myrcene	988	991 ± 2	0.46 ± 0.06	0.23 ± 0.10	n.d.	n.d.	n.d.	0.01 ± 0.01	n.d.	0.27 ± 0.27
**13**	10.56	Hexanoic acid ethyl ester	998	1000 ± 2	0.34 ± 0.06	0.01 ± 0.01	n.d.	n.d.	n.d.	n.d.	n.d.	n.d.
**14**	10.68	Octanal	1003	1003 ± 2	0.26 ± 0.05	0.71 ± 0.15	0.19 ± 0.03	2.08 ± 0.66	0.13 ± 0.04	0.23 ± 0.03	0.20 ± 0.02	1.43 ± 0.34
**15**	10.8	3-Carene	1007	1011 ± 2	0.44 ± 0.15	0.70 ± 0.17	0.02 ± 0.02	0.05 ± 0.05	n.d.	0.23 ± 0.20	n.d.	0.24 ± 0.24
**16**	11.25	*o*-Cymene	1023	1022 ± 2	0.49 ± 0.06	0.36 ± 0.14	n.d.	0.04 ± 0.04	0.18 ± 0.01	0.02 ± 0.01	n.d.	n.d.
**17**	11.38	Limonene	1028	1030 ± 2	2.09 ± 0.24	0.006 ± 0.011	0.11 ± 0.03	n.d	0.13 ± 0.05	0.16 ± 0.14	n.d.	n.d.
**18**	11.57	*cis*-β-Ocimene	1035	1038 ± 2	0.29 ± 0.04	0.008 ± 0.010	n.d.	n.d.	0.04 ± 0.03	n.d.	n.d.	n.d.
**19**	12.18	γ-Terpinene	1059	1060 ± 3	0.27 ± 0.03	0.41 ± 0.21	n.d.	n.d.	n.d.	n.d.	n.d.	n.d.
**20**	12.9	α-Terpinolene	1084	1088 ± 2	0.59 ± 0.15	0.86 ± 0.21	n.d.	0.14 ± 0.09	0.07 ± 0.04	0.01 ± 0.01	n.d.	n.d.
**21**	13.12	1-Allyl-2-isopropyldisulfane	1093	1076 ± 23	0.68 ± 0.06	0.73 ± 0.19	0.33 ± 0.03	0.11 ± 0.11	0.39 ± 0.05	0.49 ± 0.03	0.23 ± 0.03	0.20 ± 0.12
**22**	13.42	Nonanal	1104	1104 ± 2	2.08 ± 0.26	8.66 ± 2.16	0.86 ± 0.08	10.68 ± 1.50	0.51 ± 0.19	1.55 ± 0.24	1.00 ± 0.10	11.15 ± 1.51
**23**	13.51	Disulfide, propyl	1108	1107 ± 8	43.42 ± 2.02	35.85 ± 7.41	55.92 ± 1.45	14.46 ± 2.59	56.61 ± 3.05	42.16 ± 3.28	35.33 ± 2.56	17.55 ± 6.43
**24**	13.7	(*E*)-1-(Prop-1-en-1-yl)-2-propyldisulfane	1115	1118 ± n/a	6.67 ± 1.30	9.16 ± 3.65	10.55 ± 0.68	6.80 ± 0.97	13.93 ± 0.93	13.13 ± 0.87	35.47 ± 2.94	4.45 ± 0.77
**25**	13.83	1-((*E*)-Prop-1-en-1-yl)-2-((*Z*)-prop-1-en-1-yl)disulfane	1120	1124 ± n/a	1.48 ± 0.12	0.42 ± 0.19	2.27 ± 0.20	n.d.	1.73 ± 0.50	2.78 ± 0.24	2.01 ± 0.22	n.d.
**26**	13.97	(*E*,*E*)-Propenyl disulfide	1126	1129 ± n/a	1.13 ± 0.14	0.67 ± 0.27	2.26 ± 0.19	0.19 ± 0.13	1.47 ± 0.44	2.40 ± 0.24	3.88 ± 0.29	0.20 ± 0.20
**27**	14.15	*cis*-2-Mercapto-3,4-dimethyl-2,3-dihydrothiophene	1133	1138 ^c^	0.72 ± 0.06	2.07 ± 0.46	1.36 ± 0.11	6.68 ± 0.45	1.00 ± 0.31	1.54 ± 0.17	1.20 ± 0.10	5.06 ± 0.43
**28**	14.61	Trisulfide, methyl propyl	1151	1150 ± 7	1.50 ± 0.17	1.04 ± 0.13	0.45 ± 0.05	0.66 ± 0.27	0.35 ± 0.04	0.33 ± 0.05	0.34 ± 0.03	0.97 ± 0.42
**29**	14.78	4-Methyl-1,2,3-trithiolane	1158	1154 ± 4	0.18 ± 0.02	0.35 ± 0.19	0.53 ± 0.05	0.63 ± 0.23	0.41 ± 0.14	0.75 ± 0.14	0.19 ± 0.04	0.76 ± 0.64
**30**	14.84	(*Z*)-1-Methyl-3-(prop-1-en-1-yl) trisulfane	1160	1164 ± n/a	0.69 ± 0.05	0.61 ± 0.21	0.42 ± 0.06	1.17 ± 0.45	0.14 ± 0.03	0.17 ± 0.04	0.16 ± 0.03	0.10 ± 0.08
**31**	14.97	(*E*)-1-Methyl-3-(prop-1-en-1-yl) trisulfane	1165	1169 ± 1	0.89 ± 0.07	1.01 ± 0.21	0.58 ± 0.08	1.23 ± 0.27	0.15 ± 0.04	0.17 ± 0.03	0.17 ± 0.02	2.45 ± 0.69
**32**	15.35	*trans*-2-Mercapto-3,4-dimethyl-2,3-dihydrothiophene	1180	1185 ^c^	0.89 ± 0.11	2.74 ± 0.49	1.94 ± 0.16	8.42 ± 0.60	1.39 ± 0.39	2.08 ± 0.22	3.11 ± 0.20	6.71 ± 0.61
**33**	15.98	Decanal	1205	1206 ± 2	1.88 ± 0.23	2.37 ± 0.55	1.12 ± 0.20	3.07 ± 0.42	0.52 ± 0.17	1.93 ± 0.25	1.39 ± 0.25	4.29 ± 0.83
**34**	16.24	Tetrasulfide, dimethyl	1216	1234 ± 11	0.61 ± 0.30	1.57 ± 0.41	0.14 ± 0.02	1.82 ± 0.42	n.d.	n.d.	n.d.	1.03 ± 0.64
**35**	17.2	Disulfide, methyl 1-(methylthio)propyl	1257	1265 ± n/a	0.75 ± 0.14	0.98 ± 0.18	0.18 ± 0.02	0.06 ± 0.05	0.16 ± 0.01	0.12 ± 0.02	0.14 ± 0.01	0.09 ± 0.09
**36**	18.86	Trisulfide, dipropyl	1328	1328 ± 5	4.28 ± 0.62	2.68 ± 0.45	4.28 ± 0.71	2.27 ± 0.54	6.93 ± 1.12	6.02 ± 0.53	3.69 ± 0.33	1.82 ± 0.35
**37**	19.03	(*Z*)-1-(Prop-1-en-1-yl)-3-propyltrisulfane	1336	1331 ± n/a	2.22 ± 0.33	1.61 ± 0.30	1.17 ± 0.08	2.99 ± 0.75	1.07 ± 0.16	2.59 ± 0.26	0.75 ± 0.11	5.21 ± 1.24
**38**	19.15	(*E*)-1-Allyl-3-(prop-1-en-1-yl) trisulfane	1341	1346 ± 14	1.93 ± 0.12	1.18 ± 0.21	1.63 ± 0.10	1.30 ± 0.43	2.26 ± 0.54	3.20 ± 0.33	1.72 ± 0.22	1.00 ± 0.67
**39**	19.22	(*E*)-1-(Prop-1-en-1-yl)-3-propyltrisulfane	1344	1345 ± 6	1.90 ± 0.15	1.62 ± 0.27	0.98 ± 0.08	2.24 ± 0.23	0.69 ± 0.10	2.73 ± 0.31	0.51 ± 0.10	2.91 ± 0.22
**40**	19.34	1,3-Di((*E*)-prop-1-en-1-yl)trisulfane	1350	1348 ± n/a	0.90 ± 0.15	0.19 ± 0.09	0.66 ± 0.06	0.35 ± 0.17	0.54 ± 0.18	1.01 ± 0.14	0.42 ± 0.07	1.02 ± 0.46
**41**	19.91	5-Methyl-1,2,3,4-tetrathiane	1369	1364 ± 5	0.06 ± 0.01	0.90 ± 0.30	0.22 ± 0.05	1.48 ± 0.29	0.15 ± 0.06	0.27 ± 0.04	0.13 ± 0.03	0.59 ± 0.45
**42**	20.37	Pentasulfide, dimethyl	1402	1415 ± n/a	0.29 ± 0.04	0.11 ± 0.09	0.17 ± 0.02	0.09 ± 0.09	0.03 ± 0.02	0.05 ± 0.02	0.07 ± 0.02	0.16 ± 0.16
**43**	20.94	1-(1-(Methylthio)propyl)-2-propyldisulfane	1426	1431 ± n/a	2.27 ± 0.34	1.23 ± 0.15	0.52 ± 0.14	0.52 ± 0.26	0.60 ± 0.12	0.32 ± 0.05	0.14 ± 0.02	0.91 ± 0.46
**44**	21.26	2-hexyl-5-methyl-3(2*H*)-Furanone	1441	1446 ± n/a	2.74 ± 0.60	1.80 ± 0.53	0.01 ± 0.01	0.44 ± 0.24	1.05 ± 0.71	n.d.	n.d.	0.63 ± 0.30
**45**	22.06	4-Ethyl-2,3,5,6-tetrathiaheptane	1479	1483 ± n/a	0.59 ± 0.25	0.45 ± 0.14	0.02 ± 0.01	0.41 ± 0.18	n.d.	n.d.	n.d.	n.d.
**46**	23.9	1-(1-(Prop-1-en-1-ylthio)propyl)-2-propyldisulfane	1593	1592 ± n/a	2.08 ± 0.19	1.14 ± 0.19	1.07 ± 0.14	0.29 ± 0.19	0.72 ± 0.11	2.31 ± 0.37	0.85 ± 0.17	0.24 ± 0.24
**47**	24.05	6-Ethyl-4,5,7-trithia-2,8-decadiene	1603	1603 ± n/a	0.55 ± 0.08	0.31 ± 0.11	0.28 ± 0.05	n.d.	0.15 ± 0.02	0.38 ± 0.06	0.21 ± 0.04	n.d.
**48**	24.1	1,2,3,5-Tetrathiane, 4,6-diethyl-, *cis*-	1607	1606 ± n/a	0.79 ± 0.14	0.72 ± 0.20	0.23 ± 0.03	0.62 ± 0.26	0.15 ± 0.04	0.47 ± 0.10	0.10 ± 0.02	0.62 ± 0.40
**49**	24.14	1,2,4,5-Tetrathiane, 3,6-diethyl-*cis*-	1611	1610 ± n/a	0.62 ± 0.10	0.34 ± 0.17	0.21 ± 0.02	0.21 ± 0.12	0.13 ± 0.04	0.43 ± 0.08	0.11 ± 0.02	0.26 ± 0.20
**50**	24.24	1,2,4,5-Tetrathiane, 3,6-diethyl-, *trans*-	1619	1613 ± n/a	0.28 ± 0.03	0.02 ± 0.02	0.14 ± 0.01	n.d.	0.04 ± 0.02	0.08 ± 0.02	0.08 ± 0.01	n.d.
**51**	24.67	2-Octyl-5-methyl-3(2*H*)-Furanone	1653	1653 ± n/a	1.35 ± 0.57	1.38 ± 0.65	0.04 ± 0.01	n.d.	0.01 ± 0.01	n.d.	0.01 ± 0.01	n.d.

Exp ^a^: experimental retention indices calculated using a C7–C40 linear alkane mixture. Lit ^b^: semistandard non polar retention indices reported in the NIST14 mass spectra database [24] unless entry 27 and 32 (marked with apex “^c^”) from reference [25]. n/a: not applicable (single value available in NIST14 database). n.d.: not detected.

### 2.2. Exploratory Analysis

To obtain an overview of the relationships among the investigated onion samples and identify the major sources of variability, an exploratory analysis was performed by Principal Component Analysis (PCA). Prior to model calculation, the matrix containing the relative abundances of the volatile organic compounds (VOCs) was log-transformed (log10) to reduce the influence of highly abundant compounds and mitigate data skewness. Subsequently, autoscaling was applied to assign equal importance to all variables regardless of their absolute abundance. The resulting PCA model was described by four principal components, accounting for 61% of the total variance of the dataset (31%, 17%, 8%, and 5%, respectively). Although a larger number of principal components was required to describe the overall variability, the score and loading plots of the first two components already provided valuable information on the relationships among the different onion varieties and the compounds responsible for their differentiation.

The PCA biplot (Figure 2), combining the score and loading plots, provides a comprehensive overview of the relationships among the investigated onion samples and the volatile compounds responsible for their differentiation. The first two principal components revealed a clear organization of the samples according to geographical origin and harvest year. Although the two Fara harvests occupied the upper half of the score plot, they formed two distinct yet closely related groups. Fara 2024 is located in the positive-PC1/positive-PC2 quadrant, forming the most compact and clearly separated cluster, whereas Fara 2025 was shifted towards lower PC1 values while maintaining positive PC2 scores. This distribution suggests that the characteristic volatile fingerprint of the Fara landrace was preserved across the two growing seasons. The separation between the two harvests was not associated with the appearance or disappearance of specific volatile compounds, but rather with differences in their relative distribution within the overall volatile profile.

Interestingly, several of the variables contributing most strongly to the discrimination of the White Onion of Fara Filiorum Petri were non-sulfur compounds. In particular, monoterpenes, including β-pinene (**11**), β-myrcene (**12**), *o*-cymene (**16**), *cis*-β-ocimene (**18**), γ-terpinene (**19**) and α-terpinolene (**20**), together with ethyl hexanoate (**13**) and the furanone derivatives 2-hexyl-5-methyl-3(2*H*)-furanone (**44**) and 5-methyl-2-octyl-3(2*H*)-furanone (**51**), were located in the same region of the loading plot as the Fara 2025 samples.

These patterns suggest that the observed seasonal variability was mainly associated with differences in the relative balance among selected volatile families, while the overall chemical fingerprint of the White Onion of Fara Filiorum Petri was largely preserved.

The samples located at strongly negative PC1 values were mainly represented by the MDS PGI 2026 group together with the commercial samples labelled simply as “Italia”, which largely overlapped in the scores plot. This close proximity indicates a high degree of similarity in their volatile composition and suggests that these samples may share a common geographical origin or, more generally, similar pedoclimatic conditions and cultivation practices. Although PCA alone cannot be considered sufficient to infer geographical provenance, this hypothesis is supported by a subsequent examination of the product information provided by the manufacturer, which identified Apulia among the declared production areas of the commercial “Italia” onions. This observation is consistent with the overlap between the Italia and MDS PGI 2026 samples, although further investigations involving authenticated samples from different production areas would be necessary to confirm this hypothesis.

According to the loading plot, the negative-PC1 region was mainly associated with greater contributions from *syn*-propanethial-*S*-oxide (2), 2-methyl-2-pentenal (**4**), octanal (**14**), and the *cis*- and *trans*-isomers of 2-mercapto-3,4-dimethyl-2,3-dihydrothiophene (**27** and **32**). These compounds, together with lower contributions of the terpene-rich fraction associated with the Fara 2025 samples, were primarily responsible for the displacement of the MDS PGI 2026 and Italia groups along the negative direction of PC1. In particular, the greater contribution of sulfur-containing thiophenes and mercaptodihydrothiophenes suggests differences in the distribution of secondary sulfur metabolites rather than in the overall abundance of sulfur compounds, further supporting the hypothesis that origin-dependent variations mainly involve the relative balance among specific volatile constituents rather than qualitative changes in the volatilome.

The majority of the remaining onion samples, namely MDS, MDS PGI 2025, Calabria, and Giarratana, were distributed in the negative-PC2 region of the score plot. Their position was mainly associated with greater contributions from sulfur-containing compounds, particularly (*E*)-1-(prop-1-en-1-yl)-2-propyldisulfane (**24**), 4-methyl-1,2,3-trithiolane (**29**), dipropyl trisulfide (**36**), 1,3-di((*E*)-prop-1-en-1-yl)trisulfane (**40**) and 5-methyl-1,2,3,4-tetrathiane (**41**). These compounds are representative degradation products of onion sulfur precursors [26,27].

Although these four groups occupied the same general region of the score plot, they could still be distinguished by their relative distribution along PC1 and PC2. Giarratana formed the most compact cluster at slightly positive PC1 and negative PC2 values, whereas Calabria was displaced towards the highest positive PC1 scores. MDS was located in a lower PC1 region, while MDS PGI 2025 occupied an intermediate position between MDS and Calabria. This arrangement suggests that, despite sharing a common sulfur-rich volatilomic profile, each cultivar was characterized by a distinct balance among the sulfur compounds contributing to the observed variability.

Interestingly, a marked year-related pattern was also observed for the MDS PGI onions. While the 2025 harvest clustered together with the other negative-PC2 groups and was mainly associated with higher-molecular-weight sulfur compounds, the 2026 harvest shifted towards strongly negative PC1 values, where it overlapped with the Italia. This pronounced displacement suggests that seasonal effects may have influenced the relative balance of sulfur metabolites to a greater extent in the MDS PGI onions than in the White Onion of Fara Filiorum Petri, whose two harvests remained closely grouped despite the observed quantitative differences.

Overall, the PCA biplot shows that the observed differentiation among the investigated onion varieties was primarily associated with differences in the relative distribution of individual sulfur compounds, while aldehydes, monoterpenes, and furanone derivatives provided an additional contribution to the multivariate sample distribution. Moreover, the close proximity of the two Fara harvests confirms the year-to-year consistency of the characteristic volatile fingerprint of the White Onion of Fara Filiorum Petri despite seasonal variability.

### 2.3. Authentication of White Onion of Fara Filiorum Petri by Soft Independent Modeling of Class Analogy (SIMCA)

To develop and validate the authentication model, the complete dataset, consisting of 84 onion samples, was divided into a calibration (training) set and an external test set using the Duplex algorithm [28], ensuring a representative distribution of the samples throughout the multivariate space. Twenty samples were assigned to the external test set, while the remaining 64 samples were used for model calibration.

Since the objective of this study was the authentication of the White Onion of Fara Filiorum Petri, the classification problem was formulated as a two-class problem consisting of Class Fara and Class Others. Class Fara included all authentic White Onion of Fara Filiorum Petri samples, whereas Class Others comprised all the remaining Italian white onion varieties investigated in this work. The Fara class consisted of 22 samples in the training set and 9 samples in the external test set, whereas the Others class comprised 42 training samples and 11 external test samples. The SIMCA model was therefore developed on Class Fara. To evaluate the influence of data preprocessing on model performance, four different preprocessing strategies were investigated: mean-centering (MC), autoscaling (AS), log10 transformation followed by mean-centering (log10 + MC), and log10 transformation followed by autoscaling (log10 + AS). For each preprocessing strategy, the optimal number of principal components was determined by maximizing the classification efficiency during seven-fold cross-validation. Classification efficiency was calculated as the geometric mean of sensitivity and specificity. The efficiencies obtained for the optimal models were 68.98%, 56.39%, 79.77% and 80.18% for MC, AS, log10 + MC and log10 + AS, respectively, using 6, 6, 6 and 3 principal components. Since log10 transformation followed by autoscaling achieved the highest cross-validation efficiency while requiring the lowest number of principal components, this preprocessing strategy was considered the most parsimonious and was therefore selected for the final authentication model. The predictive ability of the selected SIMCA model was subsequently evaluated using the external test set. The model correctly recognized 8 of the 9 authentic White Onion of Fara Filiorum Petri samples, corresponding to a sensitivity of 88.9%, while all non-Fara samples were correctly rejected, yielding a specificity of 100%. These results demonstrate the capability of the proposed SIMCA model to discriminate authentic White Onion of Fara Filiorum Petri from other Italian white onion varieties, with only one false negative and no false positive classifications.

The T^2^/Q plot of the optimal SIMCA model is shown in Figure 3. The plot illustrates the distribution of both calibration (empty symbols) and external validation (filled symbols) samples with respect to the Class Fara model. The dashed line represents the acceptance boundary defining the multidimensional space occupied by authentic White Onion of Fara Filiorum Petri samples. Most Fara samples are located within the model boundaries and formed a relatively compact distribution, indicating that the selected principal components adequately described the natural variability of the target class. The overlap between calibration and external validation samples further demonstrates that the model successfully captures the intrinsic characteristics of authentic Fara onions. Only one external Fara sample fell outside the acceptance region, corresponding to the single false negative classification reported in the external validation. In contrast, the samples belonging to the Class Others were mainly distributed outside the acceptance region, confirming that their volatile profiles differed sufficiently from those of the authentic Fara onions. This finding is particularly relevant for authenticity applications, where minimizing false positive classifications is essential to prevent non-authentic products from being incorrectly recognized as genuine. Overall, the T^2^/Q plot confirms the good discriminatory ability of the proposed SIMCA model. The compact distribution of the Fara samples, together with the complete rejection of the non-target onions, demonstrates that the volatile fingerprint obtained by HS-SPME-GC-MS provides sufficient chemical information to build a one-class authentication model for the White Onion of Fara Filiorum Petri.

## 3. Materials and Methods

### 3.1. Samples

The samples analyzed in this study consisted of individual onion bulbs and did not include composite samples. Different bulbs were considered biological replicates. Whenever multiple chromatograms were obtained from the same bulb, they derived from independent analyses of different portions of that bulb, prepared and stored as described in Section Samples Storage and Preparation for the Analysis.

- Flat White Onion from Fara Filiorum Petri (Abruzzi Region, Italy), harvested in 2024 and 2025 (FFP_2024 and FFP_2025), supplied directly by the Associazione Produttori Cipolla Bianca Piatta di Fara Filiorum Petri (production consortium). The geographical origin was verified through the producer’s documentation. For FFP_2024, five individual bulbs were analyzed, yielding 19 GC-MS chromatograms. For FFP_2025, 12 individual bulbs were analyzed, yielding 12 GC-MS chromatograms.
- White Onion from Margherita di Savoia (Apulia Region, Italy; PGI product), harvested in 2025 and 2026 and purchased from a supermarket in L’Aquila (MDS_PGI_2025 and MDS_PGI_2026). The product packaging clearly reported the denomination “Cipolla Bianca di Margherita IGP”, which was used to verify the declared geographical origin. For MDS_PGI_2025, five individual bulbs were analyzed, yielding 12 GC-MS chromatograms. For MDS_PGI_2026, 4 individual bulbs were analyzed, yielding 9 GC-MS chromatograms.
- White Onion from Margherita di Savoia (Apulia Region), harvested in 2025 and purchased from a greengrocer in San Severo (Foggia, Italy) (MDS_2025). The origin was declared by the retailer. Five individual bulbs were analyzed, yielding 11 GC-MS chromatograms.
- White Onion from the Calabria Region, harvested in 2025 and purchased from a supermarket (CAL_2025). The origin was declared by the retailer. Two individual bulbs were analyzed, yielding 5 GC-MS chromatograms.
- White Onion from Giarratana (Sicily Region), harvested in 2025 and purchased from a greengrocer in L’Aquila (GIARR_2025). The origin was declared by the retailer. Four individual bulbs were analyzed, yielding 11 GC-MS chromatograms.
- White Onion purchased from a supermarket in L’Aquila and labeled only as originating from Italy (ITA_2026). No more specific geographical indication was available on the commercial label. 5 individual bulbs were analyzed, yielding 5 GC-MS chromatograms.

#### Sample Storage and Preparation for the Analysis

Upon arrival at the laboratory, the dry outer skin was removed from each onion bulb. The bulbs were cut into small pieces, individually packaged in sealed bags, and frozen at −20 °C. All samples were stored frozen for at least one week before the analysis. No freeze–thaw cycles were applied. Each frozen onion portion was thawed only once immediately before analysis. Prior to HS-SPME-GC-MS analysis, frozen onion samples were allowed to thaw at room temperature for a few minutes, finely chopped, and gently blotted with filter paper to remove excess surface moisture. Approximately 600 mg of freshly prepared onion tissue were immediately transferred into a 4 mL glass vial and sealed with a Mininert^®^ valve cap (Sigma-Aldrich, Saint Louis, MO, USA). To ensure reproducibility, the time interval between sample preparation, vial sealing, and the start of the HS-SPME analysis was minimized and kept as constant as possible for all measurements. Each GC-MS measurement was performed on a freshly prepared sample portion; when multiple analyses were obtained from the same bulb, independently prepared portions were used. The sealed vial was then placed in the thermostated oil bath and subjected to HS-SPME analysis under the conditions described below.

### 3.2. HS-SPME-GC-MS Analysis

Volatile compounds were extracted by headspace solid-phase microextraction (HS-SPME) using a 100 μm polydimethylsiloxane (PDMS)-coated fiber (Supelco, Bellefonte, PA, USA). The glass vial sealed with a Mininert^®^ valve cap containing ca 600 mg of sample (see Section Samples Storage and Preparation for the Analysis) was maintained at 45 °C in an oil bath and volatile compounds were extracted by exposing the PDMS fiber to the vial headspace for 15 min. After extraction, the fiber was retracted into the needle and immediately transferred to the GC injector, where thermal desorption was performed at 250 °C for 3 min. Following each analysis, the fiber was reconditioned in the GC injector at 280 °C for 15 min. A blank run was subsequently carried out to verify the absence of carry-over effects.

GC-MS analyses were performed using a Trace 1300 gas chromatograph coupled to an ISQ LT mass spectrometer (Thermo Fisher Scientific, Waltham, MA, USA). The instrument was equipped with a split/splitless injector fitted with a dedicated SPME liner. Desorption was carried out in split mode using a split ratio of 5:1. Chromatographic separation was achieved on a J&W HP-5MS capillary column (30 m × 0.25 mm i.d., 0.25 μm film thickness; Agilent Technologies, Santa Clara, CA, USA). Helium (purity 99.9995%) was used as carrier gas at a constant flow rate of 1.1 mL min^−1^.

The oven temperature program was as follows: 40 °C for 3 min, increased to 150 °C at 6 °C min^−1^ and held for 0.5 min, then raised to 270 °C at 15 °C min^−1^ and held for 1.17 min. The ISQ LT single-quadrupole mass spectrometer was operated under the following conditions: electron ionization (EI) in positive mode at an ionization energy of 70 eV, a mass scan range of *m*/*z* 25–700, a scan cycle time of 0.2 s, a transfer line temperature of 270 °C, and an ion source temperature of 280 °C. GC-MS data processing was performed using Xcalibur software (version 4.1; Thermo Fisher Scientific, Waltham, MA, USA).

Volatile compounds were tentatively identified by comparison of their mass spectra with those contained in the NIST 14 mass spectral library [24] and by comparison of their linear retention indices with literature data. Only compounds showing a NIST match factor higher than 750 were considered; moreover, all assignments were manually verified through inspection of the mass spectra and evaluation of their plausibility based on previous reports of onion volatiles. Experimental RIs were calculated using a homologous series of n-alkanes (Retention Index Standard aliphatic C7–C40 hydrocarbons dissolved in hexane—from Sigma-Aldrich, Saint Louis, MO, USA) analysed under the same chromatographic conditions. RI agreement was considered acceptable when the experimental value fell within the literature RI uncertainty range (±1 RI unit tolerance) or, when only a single literature RI value was available, within ±6 RI units. Relative abundances were expressed as percentage peak areas obtained from the total ion chromatograms.

### 3.3. Exploratory Analysis

Exploratory data analysis (EDA) comprises a set of statistical and graphical techniques aimed at obtaining a preliminary understanding of complex datasets. In chemometrics, exploratory methods are widely employed to investigate the structure of the data, identify patterns and trends, and highlight similarities and differences among samples and variables. These approaches are particularly useful for complex datasets such as volatile profiles, where a large number of correlated variables may mask the underlying data structure and hinder direct interpretation. Among exploratory techniques, Principal Component Analysis (PCA) [29] is one of the most commonly used methods because it enables dimensionality reduction while retaining most of the information contained in the original dataset.

PCA is an unsupervised multivariate projection method that decomposes the data matrix, X (N×M), into the product of two lower-dimensional matrices according to

(1) $$X=TP^{T}+E$$

where T (N×F) is the score matrix containing the coordinates of the samples in the reduced latent-variable space, P (F×M) is the loading matrix describing the contribution of the original variables to each principal component, and E is the residual matrix containing the unexplained variation. The principal components are sequentially extracted so as to maximize the variance explained by the model under the constraint of orthogonality. The number of principal components was selected by considering the Root Mean Square Error in Cross-Validation (RMSECV) together with the additional variance explained by successive components. By projecting samples onto a low-dimensional space defined by the first principal components, PCA facilitates the visualization of relationships among samples, the identification of natural groupings and trends, and the interpretation of the variables responsible for the observed patterns through the combined examination of score and loading plots.

In the present study, PCA was applied to the matrix containing the relative abundances of the tentatively identified volatile organic compounds (VOCs) to investigate the overall structure of the dataset, explore differences among onion samples, and identify the compounds responsible for the characteristic volatile fingerprint of the White Onion of Fara Filiorum Petri in comparison with other Italian white onion varieties.

### 3.4. Soft Independent Modeling of Class Analogy (SIMCA)

Soft Independent Modeling of Class Analogy is a supervised one-class classification technique based on Principal Component Analysis [30,31]. Unlike discriminant classification methods, which simultaneously model all predefined classes, SIMCA constructs an independent PCA model exclusively for the class of interest. Consequently, each sample can be characterized by two complementary statistics: the Hotelling’s T^2^ statistic, which measures the distance of the sample within the PCA model (score space), and the Q residual (orthogonal distance from the model plane). For each sample, both T^2^ and Q statistics are evaluated simultaneously to determine whether its multivariate profile is compatible with that of the modelled class. Samples exhibiting both low score distances and low residuals are considered consistent with the class model, whereas large values of either statistic indicate that the sample deviates from the modeled class and should therefore be rejected. The simultaneous evaluation of T^2^ and Q is commonly visualized by means of a T^2^/Q plot, where the acceptance region defines the multidimensional boundary of the target class. Following the approach proposed by Qin, the normalized T^2^ and Q statistics were combined into a single acceptance criterion using a threshold equal to $\sqrt{2}$. Accordingly, samples with a combined normalized distance lower than this value are accepted as belonging to the modeled class, whereas samples exceeding this limit are rejected as non-members. This criterion accounts simultaneously for both the leverage of a sample within the PCA model and its residual distance from the model, providing a balanced assessment of class membership. Because SIMCA models only the target class, it is particularly suitable for food authentication studies, where the objective is to verify the authenticity of a product while rejecting all non-authentic samples, rather than assigning unknown samples to one of several predefined categories.

The performance of the SIMCA models was evaluated in terms of sensitivity, specificity and efficiency. Sensitivity represents the proportion of authentic samples correctly accepted by the model, whereas specificity corresponds to the proportion of non-target samples correctly rejected. Overall classification performance was assessed by the efficiency, calculated as the geometric mean of sensitivity and specificity. During model optimization, these figures of merit were estimated by seven-fold cross-validation and used to select the optimal preprocessing strategy and the appropriate number of principal components. The predictive performance of the selected model was subsequently assessed on the independent external test set.

All chemometric analyses were performed using in-house routines developed in MATLAB R2015b (The MathWorks, Inc., Natick, MA, USA).

## 4. Conclusions

This study provides the first in-depth profiling of the volatile fraction extracted via HS-SPME of the Flat White Onion of Fara Filiorum Petri. A total of 51 volatile organic compounds were tentatively identified, confirming the predominance of sulfur-containing compounds typical of *Allium cepa* while highlighting distinctive compositional features of this traditional landrace. In particular, the Flat White Onion of Fara Filiorum Petri exhibited a more diversified volatilome than the other Italian white onion varieties investigated, characterized by a lower relative abundance of sulfur compounds together with a greater contribution of terpenes, aldehydes and selected furanone derivatives. Exploratory chemometric analysis demonstrated that the Flat White Onion of Fara Filiorum Petri possesses a characteristic volatilomic fingerprint clearly distinguishable from those of the other onion varieties. Moreover, the close clustering of samples collected in two consecutive harvest years indicated that seasonal variability mainly influenced the relative abundance of specific volatile compounds while preserving the overall chemical identity of the landrace. The one-class SIMCA model further demonstrated the potential of volatile profiling for authenticity assessment. The selected model correctly recognized almost all authentic Fara samples while completely rejecting the non-target onion varieties, confirming the suitability of HS-SPME-GC-MS combined with chemometric analysis as a suitable strategy for food authentication. Overall, the present work contributes to the scientific characterization of the Flat White Onion of Fara Filiorum Petri and provides a valuable analytical basis for its authentication, conservation and valorisation.

## Figures and Tables

**Figure 1:**
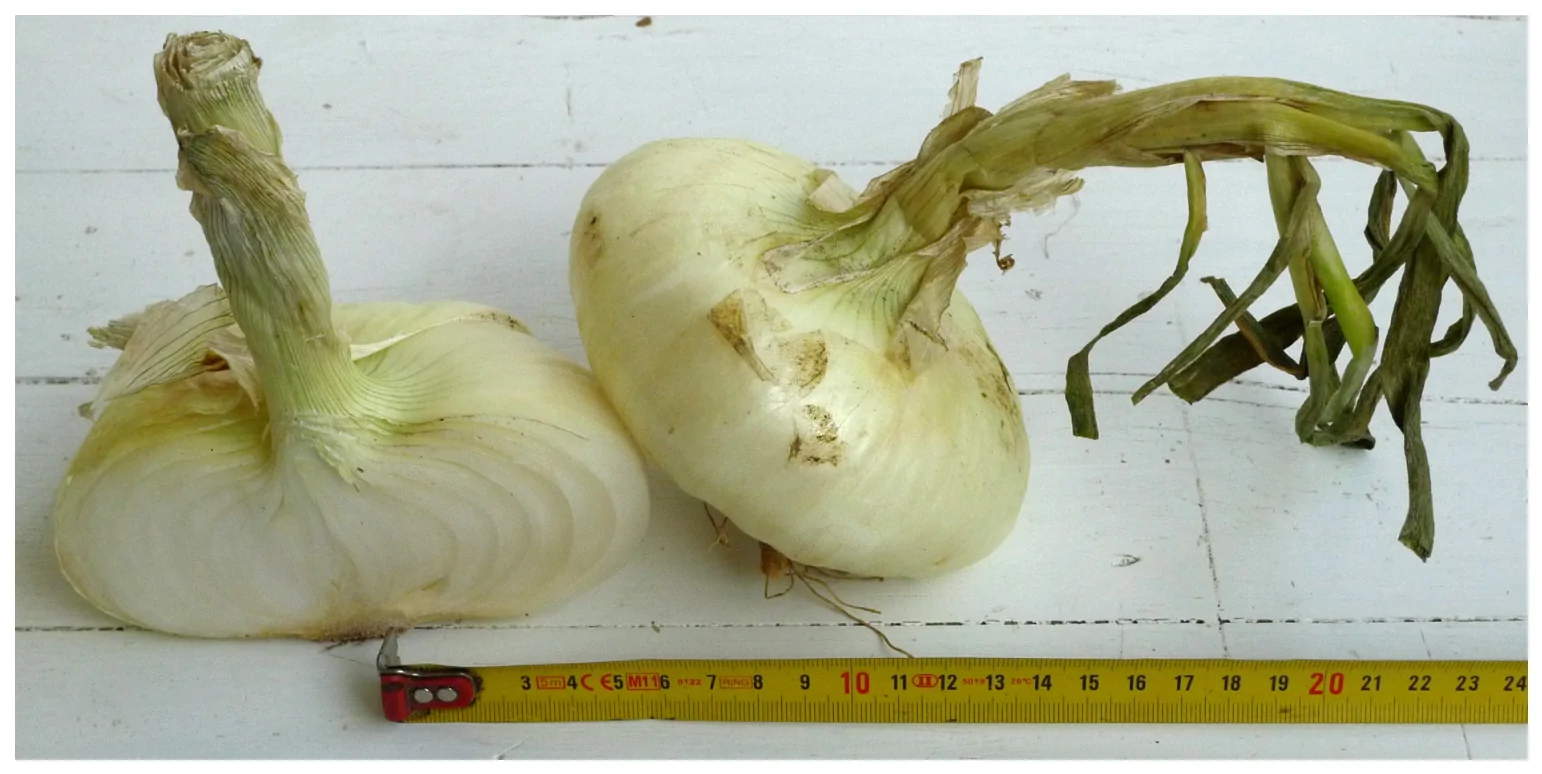
Sample of a Flat White Onion of Fara Filiorum Petri.

**Figure 2:**
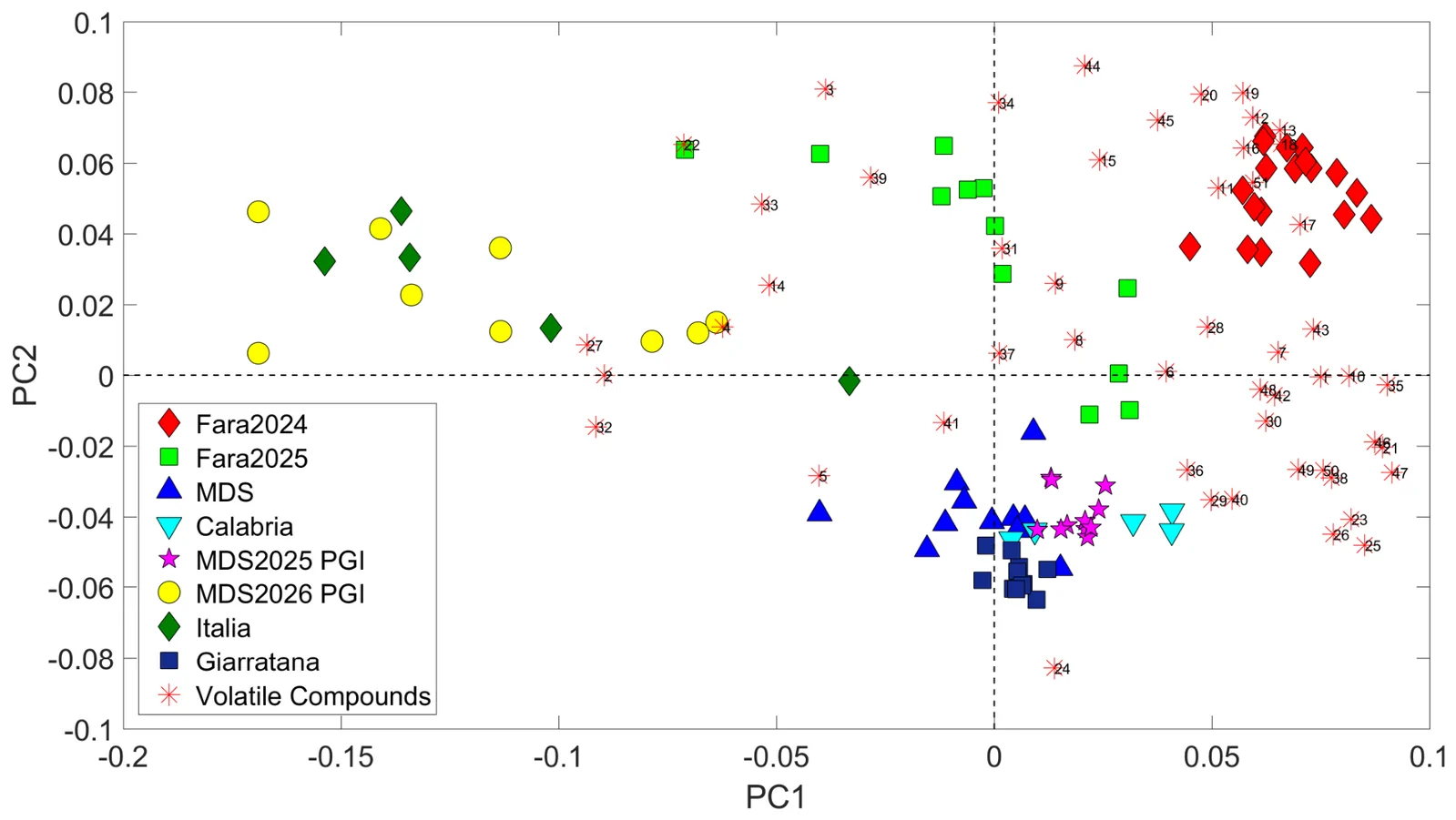
PCA biplot. Legend: Fara 2024: red diamonds; Fara 2025: green squares; MDS: blue triangles; Calabria: downward triangles; MDS PGI 2025: magenta stars; MDS PGI 2026: yellow dots; Italia: dark green diamonds; Giarratana: dark blue squares. Volatile compounds are represented by red asterisks. PC1 and PC2 explain 31% and 17% of the variance, respectively.

**Figure 3:**
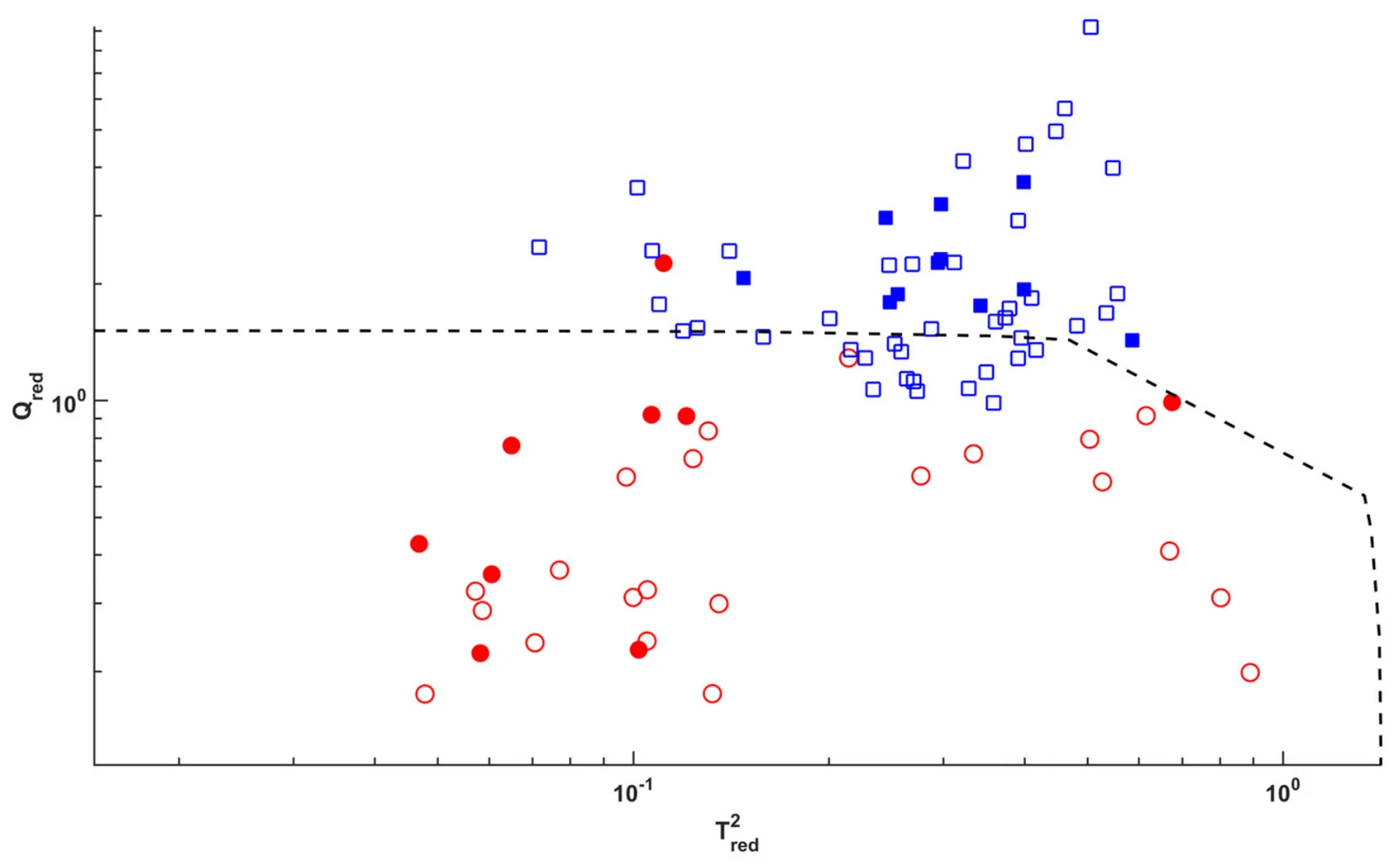
T^2^/Q plot of the optimal SIMCA model developed for the authentication of White Onion of Fara Filiorum Petri. The dashed line represents the acceptance boundary of the Fara class model. Red symbols correspond to authentic White Onion of Fara Filiorum Petri samples, whereas blue symbols represent Class Others. Empty symbols indicate calibration (training) samples and filled symbols indicate external test samples.

**Figure A1:**
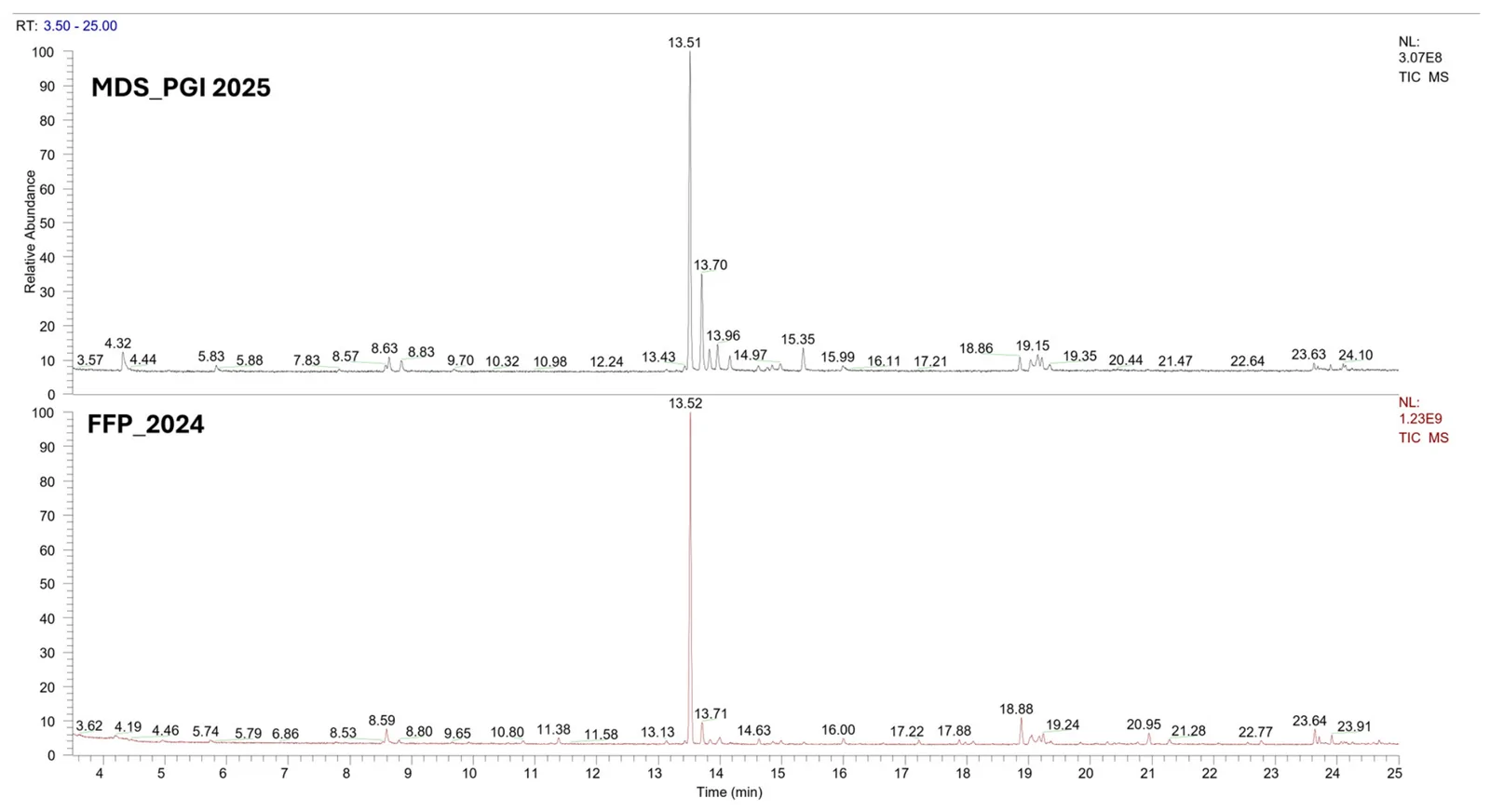
Typical HS—SPME GC MS chromatogram obtained from MDS_PGI harvest 2025 (**top**) and FFP harvest 2024 (**bottom**).

## Data Availability

Data available on request.
